# Electrochemical immunosensor with surface-confined probe for sensitive and reagentless detection of breast cancer biomarker

**DOI:** 10.1039/d0ra01192d

**Published:** 2020-06-10

**Authors:** Huage Zhong, Chang Zhao, Jie Chen, Miao Chen, Tao Luo, Weizhong Tang, Junjie Liu

**Affiliations:** Affiliated Tumor Hospital of Guangxi Medical University 71 Hedi Road Nanning 530021 PR China tangweizhong@gxmu.edu.cn liujunjie186@163.com

## Abstract

Sensitive and reliable detection of tumour markers is of great significance for early diagnosis and monitoring recurrence of cancers. Herein, a simple electrochemical immunosensor is developed with an integrated electrochemical probe on the sensing surface, which is able to sensitively and reagentlessly detect the breast cancer biomarker, human epidermal growth factor receptor 2 (ErbB2). Ferrocene (Fc) is chosen as the signal indicator and covalently grafted on cationic polyelectrolyte poly(ethylene imine) (Fc-PEI). The redox Fc-PEI could alternately assemble with carboxyl functionalized single-walled carbon nanotubes (SWNTs) on an indium tin oxide electrode through layer-by-layerelectrostatic assembly. After Anti-ErbB2 antibody is covalently immobilized onto the outermost SWNTs layer followed by blocking the electrode with bovine serum albumin, a sensing interface with recognitive probe and electrochemical probe is obtained. In the presence of ErbB2, the formed antigen–antibody complex makes a barrier to inhibit electro-transfer of inner Fc, leading to a decreased electrochemical response. Owing to the SWNTs-facilitated charge transfer and abundant surface-bound probes, the developed sensor demonstrates outstanding performance for reagentless detection of ErbB2 in terms of wide detection range (1.0–200.0 ng mL^−1^) and low detection limit (0.22 ng mL^−1^). The developed immunosensor also exhibits good selectivity, reproducibility and stability. Real analysis of ErbB2 in human serum samples is also demonstrated.

## Abstract 2

An electrochemical immunosensor with surface-confined redox probes as signal indicators was developed *via* LBL self-assembly technique for sensitive and reagentless detection of ErbB2.

## Graphical abstract

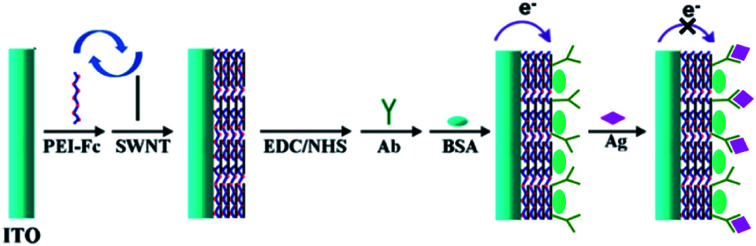

## 1. Introduction

Early diagnosis of cancers is crucial for providing the appropriate treatment processes and increasing the survival rate. Sensitive and reliable detection of low-levels of tumour markers is of great significance in early diagnosis and monitoring recurrence of cancers.^1,2^ Human epidermal growth factor receptor 2 (ErbB2), as an important tumour biomarker,^3–5^ is over-expressed in around 20–25% of invasive breast cancers and plays a major role in promoting breast cancer cell proliferation and malignant growth.^6,7^ Currently, several methods have been used for diagnostic tests of ErbB2, including enzyme-linked immuno-sorbent assay and *in situ* fluorescent hybridization.^3,8^ However, most of these techniques provide only semi-qualitative results separating patients into HER2 positive and HER2 negative. Moreover, they are usually complicated and time-consuming, need exhaustive sample pre-treatment, and require specially trained personnel to perform the complex procedures. In comparison to these techniques, electrochemical biosensors have received particular attention because of high selectivity and sensitivity, simple and rapid detection, and no need of expensive and complex instruments.^9–13^ In addition, easy integration and miniaturization of electrochemical sensors exhibit potential in point-of-care test devices.^14^

Although several electrochemical immunosensors have been reported for the detection of ErbB2,^15–18^ most detection requires external probe to produce electrochemical signals (*e.g.*, enzymatic substrates or Ag^+^ probes for stripping voltammetry or electrochemical redox probes). However, the use of solution-phase probes may compromise the detection efficiency due to diffusion limit and may bring side-effects (*e.g.* contamination to the target system) especially for long-time or repetitious detections.^19–23^ In addition, the complicated operation is not beneficial for the construction of integrated and miniaturized sensors. To tackle these problems, development of reagentless electrochemical immunosensors with surface-confined probe is of great significance.

In this work, we demonstrate an electrochemical immunosensor with surface-confined electrochemical probe for sensitive and reagentless detection of breast cancer biomarker ErbB2. Multilayers with ferrocene (Fc) as electrochemical signal indicator and single-walled carbon nanotubes (SWNTs) as conductive enhancement materials were fabricated on an indium tin oxide electrode *via* layer by layer self-assembly. Immunosensing interface was constructed by covalent immobilization of anti-ErbB2 antibodies on the SWNTs at the outmost layer of the self-assembled film. Owing to the electronic wire action of nanotubes and abundant surface-bound probes, the developed sensor demonstrates outstanding performance for reagentless detection of ErbB2 in terms of wide detection range, low detection limit, good selectivity, reproducibility and stability.

## 2. Experimental

### 2.1 Materials and reagents

ErbB2 and rabbit anti-ErbB2 monoclonal antibody (ErbB2-Ab) were purchased from Beijing ACROBiosystems Co. Ltd. (China). Poly(ethylene imine) (PEI), ferrocenecarboxaldehyde (98%), *N*-(3-dimethylaminopropy)-*N*′-ethylcarbodiimide hydrochloride (EDC), *N*-hydroxysuccinimide (NHS), bovine serum albumin (BSA), and indium-tin-oxide (ITO) coated glass were purchased from Sigma-Aldrich. Carboxyl functionalized single-wall carbon nanotubes (SWNTs) were obtained from Shenzhen Nanotech Port Co. Ltd. (China). Fresh human serum samples were provided by the Affiliated Tumor Hospital of Guangxi Medical University. The Fc–PEI complex was synthesized according to the literature.^27^ All other chemicals and reagents were of analytical grade and used without further purification. Ultrapure water (18.2 MΩ cm) from a Milli-Q Plus system (Millipore) was used to prepare all aqueous solutions throughout the work.

### 2.2 Instrumentation

Atomic force microscopy (AFM) images were obtained on a SPI3800N microscope (Seiko Instruments, Inc.). Cyclic voltammetry (CV) and differential pulse voltammetry (DPV) measurements were performed on a CHI 660D electrochemical analyzer (Shanghai CH Instrument Company, China). A conventional three-electrode system was adopted with a modified ITO as the working electrode, Ag/AgCl electrode (saturated with KCl) as the reference electrode, and a Pt sheet (1 cm × 1 cm) as the counter electrode. The ITO electrode with a certain area (3 mm in diameter) was prepared by photolithography as previously reported.^19^ Phosphate buffered solution (PBS, 0.1 M, pH 7.0) was used as the electrolyte for all electrochemical experiments. All measurements were carried out at room temperature.

### 2.3 Fabrication of sensing interface

The preparation of electrochemical immunosensor included four steps. Firstly, a clean ITO electrode with negatively charged surface was produced. ITO electrode was ultrasonicated in ethanol/water solution (v/v = 1 : 1, saturated with NaOH) for 5 min followed by successive rinsing by acetone, ethanol and ultrapure water under ultrasonication. Secondly, the multilayers with Fc as electrochemical signal indicator and SWNTs as conductive enhancement materials were fabricated. The treated ITO electrode was alternately immersed into Fc-PEI (2 mg mL^−1^) and SWNTs (0.1 mg mL^−1^) solutions for 30 min, respectively. The obtained electrode was rinsed thoroughly with ultrapure water and dried in a nitrogen stream after each assembly. This process was repeated four times until the (Fc-PEI/SWNTs)_4_ multilayer was obtained. Thirdly, covalent immobilization of antibody was performed to construct the immuno-recognition interface. The (Fc-PEI/SWNTs)_4_ multilayer modified ITO electrode was immersed into an aqueous solution containing EDC (50 mM) and NHS (50 mM) for 2 h at room temperature to activate the carboxyl groups of SWNTs, followed by rinsing with ultrapure water. Then, the activated electrode was dipped into a solution of ErbB2-Ab (100 μg mL^−1^) in PBS (0.1 M, pH 7.0) at room temperature for 2 h. As a result, ErbB2-Ab was grafted onto the outmost SWNTs layer through the formation of covalent bonds between the –COOH groups of the SWNTs and the –NH_2_ groups of ErbB2-Ab. After rinsing with PBS (0.1 M, pH 7.0) to remove excessive and physically adsorbed ErbB2-Ab, the electrode was immersed into a BSA solution (1%) and kept for 1 h to block any possible nonspecific bonding sites. Finally, the resultant BSA blocked ITO/(Fc-PEI/SWNTs)_4_/ErbB2-Ab electrode was stored at 4 °C for detection of ErbB2.

### 2.4 Electrochemical detection of ErbB2

The prepared immunosensor was incubated with ErbB2 solution for 30 min at room temperature. After rinsing with PBS, the sensing electrode was transferred into an electrochemical cell for electrochemical measurements. CV measurements were performed by scanning from 0 V to 0.7 V at a scan rate of 50 mV s^−1^. DPV were carried out from 0.7 to 0 V under the following conditions: modulation amplitude, 25 mV; modulation time, 50 ms; potential step, 5 mV; interval time, 0.5 s.

## 3. Results and discussion

### 3.1 Preparation and characterization of the self-assembled multilayers

**Scheme 1:**
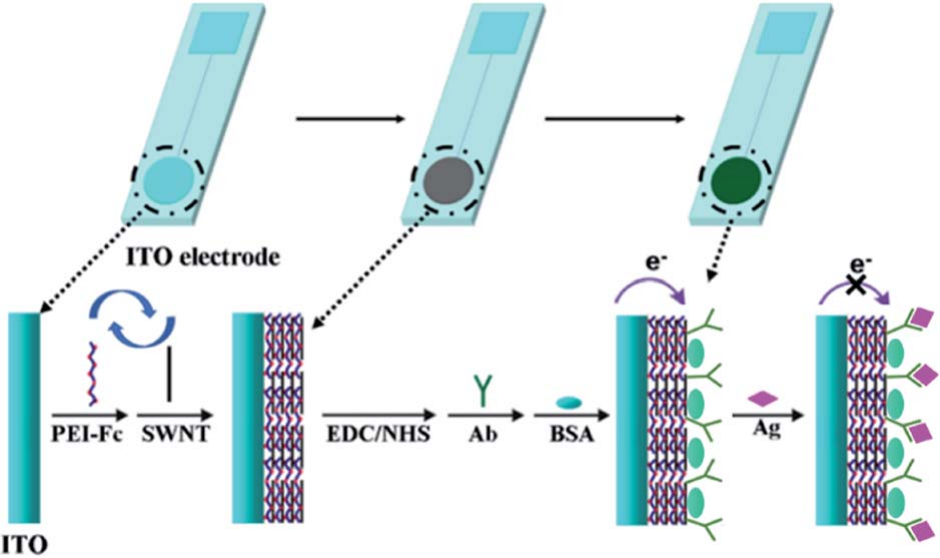
Illustration for fabrication of the immunosensor with surface-confined electrochemical probe by LBL assembly.

The preparation and detection mechanism of the immunosensor are shown in Scheme 1. As seen, through layer-by-layer (LBL) electrostatic assembly between Fc-PEI (positively charged) and carboxyl functionalized SWNTs (negatively charged), abundant Fc signal molecules are confined in Fc-PEI/SWNTs multilayers supported by ITO electrode. The immune recognition interface was constructed by the carbodiimide catalysed antibody coupling method, which has been proven to be an effective method for the covalent immobilization of active antibodies on various functional nanomaterials containing carboxyl groups.^24–26^ In addition to the four terminal amino groups in the “Y” shaped antibody molecule, the peptide chains also possess abundant free amino sites derived from special amino acid residues (*e.g.* lysine and ornithine), which can also act as binding sites for the linkage with carboxylated SWNT. Briefly, the –COOH of SWNTs activated by EDC and NHS can couple with the free –NH_2_ of anti-ErbB2 antibody (ErbB2-Ab) molecules *via* the formation of amide bond, and thus fixing the anti-ErbB2 antibodies onto the outmost SWNTs layer. The sensing interface are finally obtained after blocking nonspecific adsorption sites with BSA. When the target antigens, ErbB2 proteins, are captured by the anti-ErbB2 antibodies on the sensing interface, the formed antigen–antibody complex would increase the coverage of the electrode surface, which would insulate the interface from the electrolyte solution to some extent and therefore inhibit the electrochemical redox process of inner Fc molecules, leading to a decreased electrochemical signal.

**Fig. 1:**
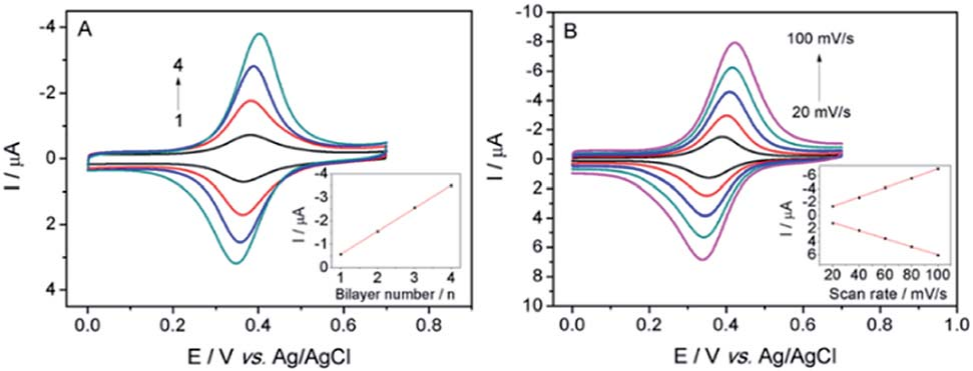
(A) CVs of ITO/(PEI-Fc/SWNTs)_*n*_ electrode with increasing the bilayer number (*n*) of PEI-Fc/SWNTs from 1 to 4 at a scan rate of 50 mV s^−1^. Inset shows the linear relationship between the anodic peak current and *n*. (B) CVs of ITO/(PEI-Fc/SWNTs)_4_ electrode at various scan rates from 20 to 100 mV s^−1^. Inset shows the linear relationship between redox peak currents and the scan rate. PBS (0.1 M, pH 7.0) is used as the supporting electrolyte.

The assembly process of Fc-PEI/SWNTs multilayers on ITO electrode was characterized by cyclic voltammetry. As shown in Fig. 1A, even one bilayer of Fc-PEI/SWNTs modified ITO exhibits a pair of well-defined redox peaks located at +0.401 V and +0.349 V, which represent the oxidation and reduction of the fixed Fc, respectively. The redox peak currents gradually increase with increasing the bilayer number of PEI-Fc/SWNTs from 1 to 4, suggesting abundant redox Fc could be controllably confined on ITO electrode through SWNTs mediated LBL assembly. A linear dependence between the peak currents and the number of the bilayer is revealed (inset of Fig. 1A), indicating the controllability of the assembling process. On the other hand, the peak-to-peak potential difference has no obvious change with increasing the number of assembly bilayer, suggesting efficient charge transfer promoted by SWNTs. In comparison with the electrode modified through MWNTs mediated LBL film, ITO/(Fc-PEI/SWNTs)_4_ exhibits lower background current signal.^28^ These advantages are benefited to the construction of electrochemical sensors with high sensitivity and low detention limit. To obtain the highest sensitivity, ITO/(Fc-PEI/SWNTs)_4_ was chosen for further investigation.

**Fig. 2:**
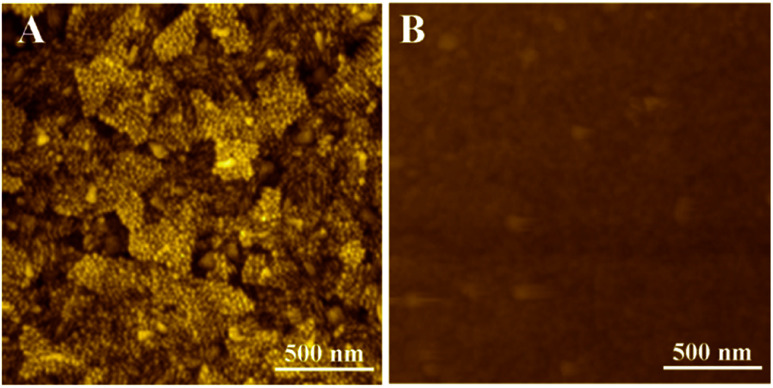
AFM images of ITO (A) and ITO/(PEI-Fc/SWNTs)_4_ electrode (B).

The effect of scan rate on the cyclic voltammograms (CVs) obtained by ITO/(Fc-PEI/SWNTs)_4_ electrode was also investigated. As shown in Fig. 1B, both the anodic and cathodic peak currents linearly increase with increasing the scan rate from 20 mV s^−1^ to 100 mV s^−1^, indicating a surface-controlled electrochemical process of the confined Fc. AFM was used to characterize the surface morphology of ITO electrode before and after assembly of (Fc-PEI/SWNTs)_4_ multilayers. As revealed in Fig. 2A, ITO electrode shows a rough surface with many uniformly distributed ITO nanoparticles, whereas no ITO nanoparticle can be seen on (Fc-PEI/SWNTs)_4_/ITO, confirming the ITO has been fully covered with the polymer-SWNTs multilayers after the LBL assembly (Fig. 2B).

### 3.2 Electrochemical characterization of the immunosensing interface

**Fig. 3:**
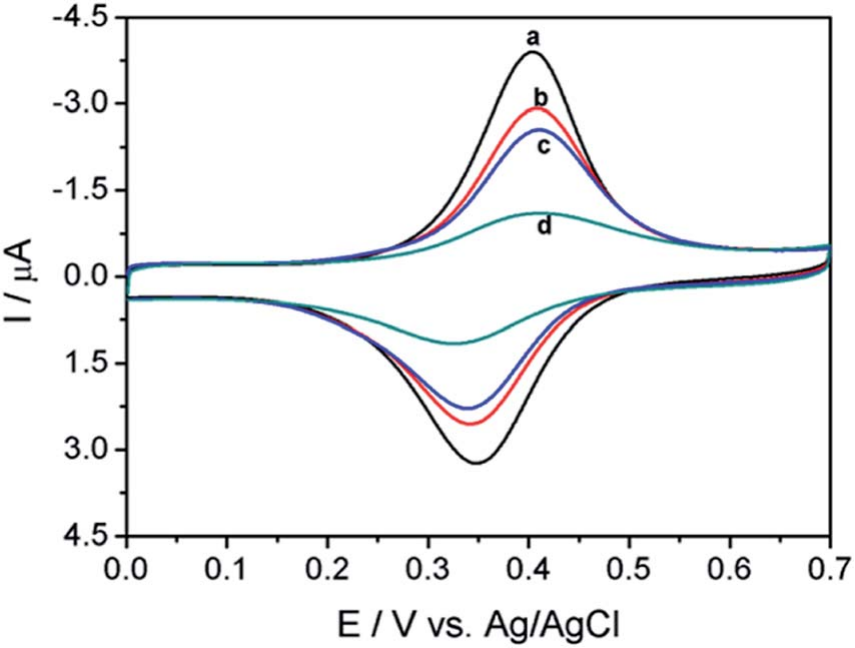
CVs of different electrodes recorded in PBS (0.1 M, pH 7.0) at a scan rate of 50 mV s^−1^. (a) ITO/(PEI-Fc/SWNTs)_4_, (b) electrode obtained after immobilization of ErbB2-Ab, (c) electrode obtained after immobilization of ErbB2-Ab followed with blocking with BSA, (d) ITO/(PEI-Fc/SWNTs)_4_/ErbB2-Ab electrode after incubation with 100.0 ng mL^−1^ ErbB2.

Electrochemical experiments were carried out to characterize the preparation of immunosensing interface and the following binding of ErbB2. As shown in Fig. 3, the ITO/(Fc-PEI/SWNTs)_4_/ErbB2-Ab electrode exhibits a decreased peak current in comparison to ITO/(Fc-PEI/SWNTs)_4_ due to the insulative properties of macromolecules. When such modified electrode is blocked by BSA, the resultant electrode displays a further reduction in peak current signal. After incubation with ErbB2 solution (100 ng mL^−1^) for 30 min, the sensing electrode presents a significant current decrease with increased peak-to-peak potential difference, suggesting the effective recognition and capture of ErbB2 by the sensing interface. As large amount of antibody–antigen complex covers the electrode surface, the electrochemical process of inner Fc probes is significantly inhibited.

### 3.3 Optimization of analytical parameters

**Fig. 4:**
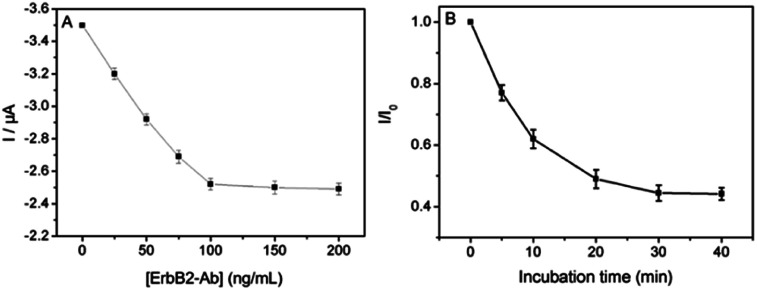
Optimization of the experimental parameters: (A) the anodic peak current change of ITO/(Fc-PEI/SWNTs)_4_ with increasing the concentration of reacted ErbB2-Ab. (B) Effect of incubation time on the DPV response of the immunosensor for ErbB2 detection.

The experimental parameters including the concentration of reacted ErbB2-Ab and immune reaction time were optimized to obtain the highest sensitivity. During optimization of the ErbB2-Ab concentration, the activated ITO/(Fc-PEI/SWNTs)_4_ electrode was incubated in ErbB2-Ab solutions at different concentrations (25, 50, 75, 100, 150, and 200 μg mL^−1^) for 2 h to evaluate the immobilization amount of ErbB2-Ab. As shown in Fig. 4A, the current signal gradually decreases with increasing the concentration of ErbB2-Ab and reaches to a plateau as the concentration is over 100 μg mL^−1^, indicating a saturated immobilization amount of ErbB2-Ab on the electrode. Therefore, the optimized concentration of ErbB2-Ab was set at 100 μg mL^−1^ for the fabrication of sensing interface. To determine the optimum immune reaction time for ErbB2 detection, kinetic experiments were carried out and the results were shown in Fig. 4B. The DPV peak current gradually decreases with increasing the reaction time and reaches a plateau as the time goes beyond 30 min due to the saturation effect. Thus, the optimal reaction time was set at 30 min.

### 3.4 Electrochemical detection of ErbB2 using the sensor

**Fig. 5:**
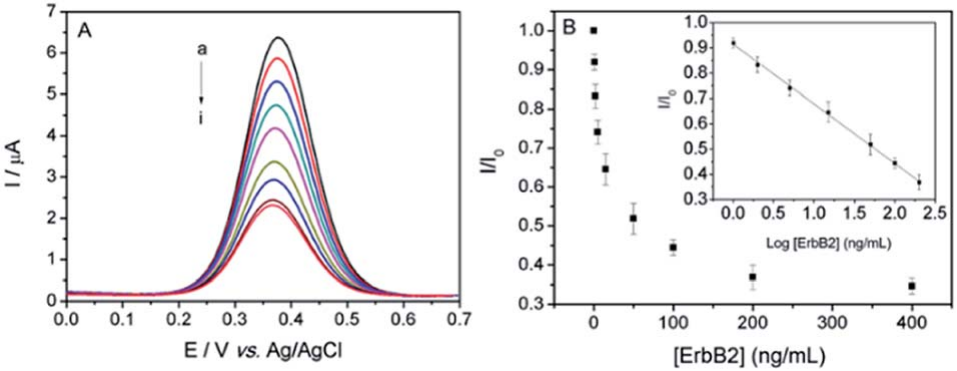
(A) DPV responses of the immunosensor towards ErbB2 at different concentrations (0, 1.0, 2.0, 5.0, 15.0, 50.0, 100.0, 200.0, 400.0 ng mL^−1^, from (a) to (i)). (B) The relative responses of the immunosensor to ErbB2 at different concentration (from 0 to 400.0 ng mL^−1^). Inset shows the calibration curve of *I*/*I*_0_*versus* logarithm of ErbB2 concentration in the range of 1.0–200.0 ng mL^−1^. The error bars represent the RSD of three measurements.

Fig. 5A displays the DPV current response of the sensor towards various concentrations of ErbB2 in PBS (0.1 M, pH 7.0). As the ErbB2 concentration increases from 1.0 ng mL^−1^ to 200.0 ng mL^−1^, the DPV peak current gradually decreases. The ratio of *I*/*I*_0_ was used as calibration, where *I*_0_ represents the original DPV peak current of ITO/(Fc-PEI/SWNTs)_4_/ErbB2-Ab, and *I* represents the peak current after incubation with ErbB2. The inset of Fig. 5B shows the corresponding calibration curve, which displays a good linearity between *I*/*I*_0_ and logarithm of ErbB2 concentration in a dynamic range of 1.0 ng mL^−1^ to 200.0 ng mL^−1^ with a correlation coefficient of 0.9991. The limit of detection (LOD) was calculated to be 0.22 ng mL^−1^ at a signal/noise ratio of 3, which is ∼68 times lower than the clinical cut-off value of ErbB2 established at 15 ng mL^−1^.^29^ On the other hand, the use of surface-confined Fc as signal indicator for reagentless detection could avoid the addition of external probes in measurement solution, providing facile and efficient strategy for detection and reducing possible contamination of the samples.

**Fig. 6:**
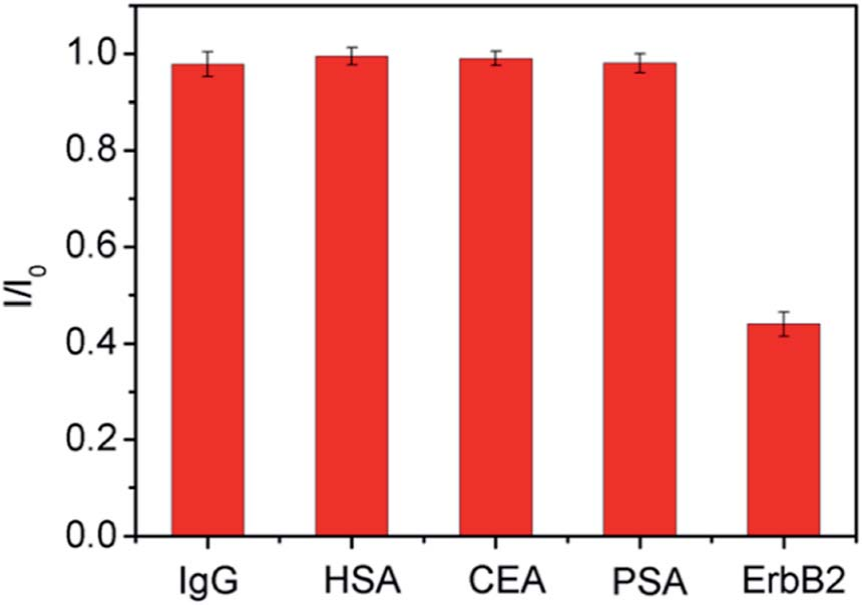
The relative responses of the immunosensor towards ErbB2 (100.0 ng mL^−1^), 1.0 μg mL^−1^ of IgG, HSA, CEA, or PSA, respectively. The error bars represent the RSD of three measurements.

The selectivity of the developed immunosensor was also investigated. The DPV current responses of the immunosensor towards ErbB2 and other different protein solutions including human immunoglobulin G (IgG), human serum albumin (HSA), carcinoembryonic antigen (CEA), or prostate specific antigen (PSA) were measured. As illustrated in Fig. 6, no obvious change of electrochemical signals is observed for even high concentration (ten times higher than that of ErbB2) of IgG, HSA, CEA, or PSA. This result suggests that the immunosensor has a good selectivity towards ErbB2 detection. To evaluate the reproducibility of the sensor, five sensing electrodes were fabricated independently for the detection of ErbB2 (100 ng mL^−1^). The relative standard deviation (RSD) of the measured currents is within 4.2%, indicating good reproducibility of the immunosensor. In order to evaluate the stability, the developed immunosensor was stored in PBS (0.1 M, pH 7.0) for 2 weeks at 4 °C. Afterwards, the detection for ErbB2 (100 ng mL^−1^) was performed. In comparison with the result obtained on the freshly prepared immunosensor, only 3.4% decrease of DPV current is observed, suggesting good stability of the immunosensor.

### 3.5 Analysis of real samples

Standard addition experiments were carried out in human serum matrices (diluted by 20 times with 0.1 M PBS, pH 7.0) to evaluate the analytical reliability and potential for practical applications of the immunosensor. Three serum samples spiked with increasing amounts of ErbB2 were analysed by the immunosensor (parallel determination of each sample for 3 times). As demonstrated in Table 1, the recoveries range from 96.7% to 103% and the RSDs are less than 4.1%. Satisfactory recoveries demonstrate good precision and potential application of the proposed immunosensor in real sample analysis.

**Table 1:** Detection of ErbB2 in human serum samples by the developed immunosensor using standard addition experiments

ErbB2 spiked (ng ml^−1^)	ErbB2 found (ng ml^−1^)	RSD (%)	Recovery (%)
5.0	4.7, 5.0, 4.8	3.2	96.7
15.0	15.3, 15.9, 15.4	2.1	107
30.0	31.1, 32.1, 29.6	4.1	103

## 4. Conclusions

In summary, electrochemical immunosensor with surface-confined redox probe is simply constructed for reagentless detection of breast cancer biomarker. Layer-by-layer assembled multilayers with ferrocene (Fc) as electrochemical signal indicator and SWNTs as conductive enhancement materials are fabricated on the supporting electrode. Owning to the presence of SWNTs and abundant confined ferrocene, the fabricated immunosensor presents good performance for the detection of ErbB2 in terms of wide detection range, low detection limit, high stability and good reliability in analysis of real samples. On the other hand, the use of surface-confined Fc as signal indicator for reagentless detection could avoid the addition of external probes in measurement solution, thus efficient reducing possible contamination of the samples. The strategy for the fabrication of immunosensors could be easily extended for the detection of other tumour biomarkers and exhibits potential in clinical diagnostics and biochemical analysis.

## Conflicts of interest

There are no conflicts to declare.

## Supplementary Material
